# Human Blood Lead Levels and the First Evidence of Environmental Exposure to Industrial Pollutants in the Amazon

**DOI:** 10.3390/ijerph16173047

**Published:** 2019-08-22

**Authors:** Thaís Karolina Lisboa de Queiroz, Karytta Sousa Naka, Lorena de Cássia dos Santos Mendes, Brenda Natasha Souza Costa, Iracina Maura de Jesus, Volney de Magalhães Câmara, Marcelo de Oliveira Lima

**Affiliations:** 1 Programa de Pós-Graduação em Epidemiologia e Vigilância em Saúde (PPGEVS), Instituto Evandro Chagas (IEC), Ananindeua 67030-000, Brazil; 2 Seção de Meio Ambiente (SAMAM), Instituto Evandro Chagas (IEC), Ananindeua 67030-000, Brazil; 3 Programa de Pós-Graduação em Ecologia Aquática e Pesca (PPGEAP), Universidade Federal do Pará (UFPA), Belem 66075-110, Brazil; 4 Programa de Pós-Graduação em Saúde Coletiva (PPGSC), Universidade Federal do Rio de Janeiro (UFRJ), Rio de Janeiro 21941-901, Brazil

**Keywords:** lead, epidemiology, total blood, ICP-MS

## Abstract

The main routes of lead (Pb) absorption are through the airways and orally, and through consumption of contaminated food and beverage, with Pb mainly being absorbed in the atmospheric particulate form. In 2012, a cross-sectional study was performed to evaluate the Pb environmental exposure in two Amazonian districts, Dom Manuel (DMN) and Laranjal (CLA), located in Barcarena City, northern Brazil. CLA is located outside the industrial area of Barcarena (control population), whereas DMN is an old community located in the vicinity of industrial activities. A significant number of residents in these districts participated in an epidemiological inquiry and blood sampling. Total Pb blood levels were quantified using inductively coupled plasma mass spectrometry (ICP-MS). The mean Pb blood level in people that live in DMN was 281.60 (98.73–570.80) μg·L^−1^, approximately nine times higher than the level found in CLA (32.77 μg·L^−1^). In these districts, the Pb blood levels showed statistically significant differences (*p* < 0.05) based on gender, schooling, residence time, and smoking. This is the first evidence of industrial environmental pollutant exposure in the Amazon.

## 1. Introduction

In the last decade, in developing countries, industrialization was directly associated with economic growth. However, most of the activities involved in industrialization were not developed by prioritizing the use of techniques and processes that are environmentally safe. Population growth and urban expansion around the areas where the industrial plants were installed were neglected. Studies showed that, around these areas, there are frequent records of the environmental impacts of exposure to contaminants generated from the emission of atmospheric pollutants and effluent discharge [1].

After the 1970s, the Pará State, northern Brazil, won international prominence due to the installation of large projects such as hydroelectrics, industries, mineral exploration, and others focused on agricultural and livestock production. In parallel, these promoted significant environmental, socioeconomic, and cultural changes in these areas. Barcarena City is an example, with industrial and port activities for alumina and aluminum ingot productions, as well as for the kaolin beneficiation processes. Around these areas, there were already established riverside, quilombola, and indigenous traditional populations. They also occupied other territories during the construction and operation stages of these enterprises. This proximity can expose the populations to toxic substances such as lead (Pb) that can be emitted from several stages of the industrialization processes [2,3,4].

In Brazil, the use of lead in gasoline was banned since the 1990s [3]. However, fossil fuels, such as mineral coal used in boilers in industrial processing of kaolinite and bauxite, emit atmospheric pollutants and generate industrial waste and effluents with high levels of trace elements, such as lead. It is also known that bauxite and kaolinite are minerals extracted from Amazonian soils that have trace elements in their geological compositions, including lead (Pb) [3]. Although the removal of these soils for mineral extraction does not occur, these elements remain immobilized in the geological material. The mineral extraction steps do, however, expose these materials to chemism and weathering. The industrial processing, involving the disaggregation of geological material through physical and chemical processes, is responsible for releasing these trace elements that are pre-concentrated in the residues and generate effluents or emit the atmospheric particulate material [4].

Pb is a toxic metal that, at room temperature, presents as a bluish-gray solid. This metal can easily be found in the earth’s crust, and its highest occurrence (86%) is in the galena ore (PbS) [5]. The main routes of absorption of lead (Pb) are through the airways and orally, and from contaminated food and beverage consumption. The main form of Pb absorbed is the atmospheric particulate form [6]. The amount of Pb absorbed in the human body may vary with particle size, solubility of the ingested compounds, and characteristics of the exposed individuals such as age, gender, nutritional status, and heredity.

Depending on the period and intensity of exposure, Pb in the blood is incorporated into calcified tissues such as bones and teeth where it can remain for 10 to 30 years and become a continuous source of endogenous contamination as it slowly leaks into the blood and soft tissues. Thus, an individual may have elevated blood Pb levels for years even after being away from the source of exposure. Therefore, total blood lead (TPb) dosages may be considered as a suitable biomarker for assessing environmental exposure to this element [6].

In this study, epidemiological and laboratory data were examined to evaluate the environmental exposure to Pb in two Amazonian populations, both located in the City of Barcarena. Laranjal (CLA) is the peripheral neighborhood of the Cabanos village. At the time of the study, it was located around 3–5 km from the industrial area, and it was initially identified as a control population. The Laranjal was created with the purpose of sheltering the population that expropriated lands during the process of implementation of the industrial complex in Barcarena.

## 2. Materials and Methods

### 2.1. Study Area

**Figure A1:**
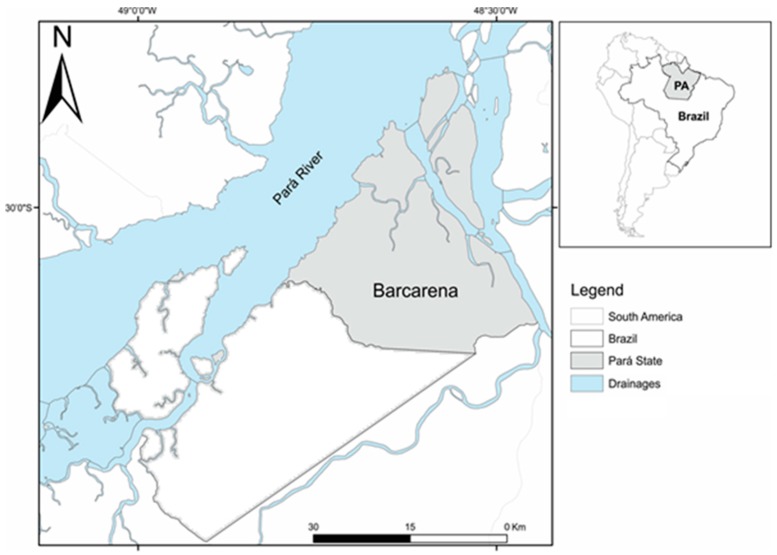
Location of the Dom Manuel (DMN) and the Laranjal (CLA) districts on the map.

**Figure A2:**
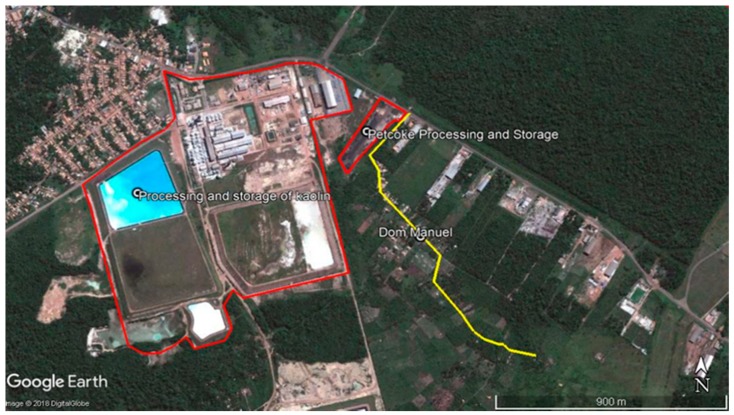
Location of the district of Dom Manuel (DMN), 2012. Source: Google Earth, 2017.

**Figure A3:**
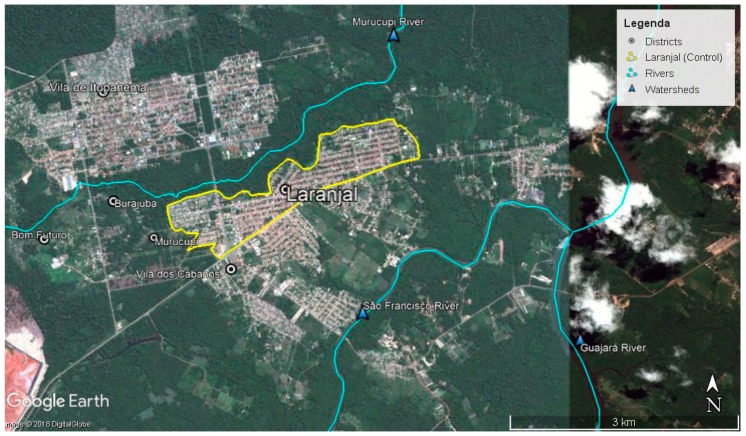
Location of the neighborhood of Laranjal (CLA), 2012. Source: Google Earth, 2017.

Barcarena is a small city in the Amazon Region (1310 km^2^) with an estimated population of 121,190 inhabitants. A port and an industrial area were installed in its territory and in the margins of the Marajó Bay in the 1970s (Figure A1). The first area selected for this study was Dom Manuel (DMN), *N* = 45, identified as potentially being exposed to metallic pollutants due to its proximity to industrial areas used for processing and storage of petroleum, and coal coke and kaolin processing (Figure A2). At the time of blood sampling, DMN residents lived in houses located 20–200 m away from industrial activities. The second selected area was Laranjal (CLA), *N* = 305, identified in this study as a control group due to it being located further from these industrial areas (Figure A3). At the time of blood sampling, CLA residents lived in houses located at a distance of 3–5 km from industrial activities.

### 2.2. Epidemiology

The study was approved by the Ethical Committee of the Evandro Chagas Institute (IEC) (0013/2008). Survey participants were selected based on geospatial distribution criteria for each assessed district, with the aim to cover homes with different distances from the industrial activities. During sampling, a population proportion of 0.5% (a larger sample size that can be calculated when previous population prevalence is unknown), precision estimate of 0.3%, and significance level of 5% were considered. In DMN, we estimated the presence of 56 individuals, and all the individuals who were in their residences were invited to voluntarily participate in this research study (*N* = 45). In Laranjal, the estimated population was 1607, and individuals were selected by street. The population evaluated had a maximum sampling error of 5% (*N* = 305).

All participants signed the informed consent form and were enrolled into the epidemiological survey for the evaluation of the characteristics of the study population. Demographics, habits, and lifestyle variables (schooling, gender, consumption of alcoholic beverages, and use of cigarettes or habit of dying the hair) were included. In addition, dietary variables (daily consumption of fruits, vegetables, red meat, fish, shellfish, or canned food) and variables related to morbidities reported by the interviewees were included. Type of housing, drinking water treatment forms (mineral water use, boiling, coagulation, use of hypochlorite, filtration, or no treatment of the water consumed), and origin of the water consumed by the interviewees were also included.

### 2.3. Sampling and Analyses

Blood samples were collected through venipuncture using disposable syringes and needles. Collected samples were stored in 4.0-mL polypropylene tubes, frozen (−20 °C), and transported in thermal boxes to the Toxicology Laboratory at Environmental Section (SAMAM) at the Evandro Chagas Institute. Before the analysis, an aliquot of 200 µL was transferred into a polypropylene tube and shaken for 30 s using vortex (Model Q220M, QUIMIS INC, Diadema, São Paulo (SP), Brazil). In this tube, 200 µL of a solution containing 1.0% acid and 0.1% Triton X-100 was added and resuspended for 30 s using vortex. After a 5-min incubation, the sample solution was centrifuged at 6000 rpm and the supernatant was withdrawn and transferred to a 15-mL Teflon tube with a screw cap. The Pb was quantified using an inductively coupled plasma mass spectrometer (ICP-MS, Brand: Bruker, Model: 820-MS) installed in a clean room environment. For quality control, certified reference materials (Seronorm^®^ Trace Elements Whole Blood Lyophilized L-2 and Seronorm^®^ Trace Elements Whole Blood Lyophilized L-1) were used [7].

The analytical quality control used for the quantification of blood Pb was 1.283 as the limit of quantification (LQ) and 0.385 as the limit of detection (LD). The mean recovery percentage of the reference materials used in the quantification of blood Pb was 90.70% for Whole Blood Lyophilized L-1 and 114.80% for Whole Blood Lyophilized L-2.

### 2.4. Statistical Analysis

Statistical analysis was performed using Microsoft Excel 2013 (Microsoft, Redmond, WA, USA), Minitab^®^ 17.0 (State College, Pensilvânia, EUA), and MedCalc (Seoul, Republic of Korea). Descriptive statistics included measures of central tendency (mean), dispersion, (standard deviation), minimum, and maximum. We also calculated the number of missing observations for each of the variables for each district evaluated. We performed the identification of possible outliers for the DMN and CLA districts. Based on the Grubbs test, the outliers found were excluded from the mean value.

The MedCalc software was used to perform the non-parametric Kruskal–Wallis statistical test. We compared the medians of blood Pb concentrations between districts and descriptive epidemiological variables, and compared the differences in Pb concentrations in the biological samples with demographics, habits and lifestyle variables, and dietary variables, considering a level of statistical significance of 95% (*p* < 0.05).

The blood Pb levels found in individuals residing in DMN and CLA districts below the LD were adjusted to half the quantification limit (2/LQ) for the statistical analyses of blood Pb levels, based on procedures used in previous studies [8].

## 3. Results

The mean blood Pb levels and the frequency in each district, excluding the two outliers, were reported for DMN (994.66 μg·L^−1^, G = 4.29, *p* = 0.000) and CLA (181.27 μg·L^−1^, G = 5.26, *p* = 0.000), where the result of the Grubbs test is given by the result of G, which is the ratio of the subtraction of the sample mean to the lowest or highest value and the standard deviation. Table A1 shows the results of the descriptive statistics. The mean blood Pb level in the total study population in DMN was 281.60 μg·L^−1^ (DP ± 120.90), while, in CLA, it was 32.77 μg·L^−1^ (DP ± 26.85). The mean blood Pb level in DMN was approximately nine times higher than that in CLA (control group).

### 3.1. Pb Blood Levels and Epidemiological Variables

Table A2 shows the mean Pb concentrations in the blood associated with some epidemiological variables. According to gender, in DMN, the blood Pb level was higher in men (290.40 μg·L^−1^). In CLA, there was no significant difference (*p* = 0.074) by gender, despite the predominance of women (54.75%).

In DMN, Pb levels were always higher than those in CLA for all age groups. In this district, despite the majority of the individuals studied (42%) being adults (20–59 years old), the mean Pb levels (317.40 μg·L^−1^) among the elderly (≥60 years) were about 12% higher than the population average (281.60 μg·L^−1^). In CLA, this observation was repeated, and Pb levels (34.98 μg·L^−1^) among the elderly were approximately 6% higher than those in the average population (32.77 μg·L^−1^).

Looking at the educational variable, the mean Pb level was higher in DMN individuals who reported only attending elementary school (311.00 μg·L^−1^), noting this group formed 60% of the study population. In CLA the mean Pb level was higher (48.10 μg·L^−1^) for individuals who were illiterate, despite the fact that the majority of respondents (40.60%) only gained elementary education.

Regarding the time of residence in the area (in years), in DMN, the mean Pb levels were higher (307.10 μg·L^−1^) for individuals that lived in the area for over 10 years, and these were the most common in this group (35.55%). For CLA, no significant differences (*p* = 0.130) were observed between residence time in the area. However, it should be noted that the majority of individuals evaluated (61.93%) lived longer than 10 years in this community.

In DMN, only 11.11% of the study population were smokers, and, within this group, the mean Pb levels (418.00 μg·L^−1^) were about 48% higher than mean levels in the total population. For CLA subjects, the mean Pb level also showed an increase for individuals who reported cigarette usage (39.90 μg·L^−1^); however, the majority of the population here (59.67%) were non-smokers.

In DMN, the frequency of alcohol consumption in the population was high (35.55%), and only about 11.11% of the individuals evaluated reported no consumption of any type of alcohol. Blood Pb levels in subjects reporting alcohol consumption (294.40 μg·L^−1^) were lower than those in non-consumers (368.30 μg·L^−1^). However, the Kruskal–Wallis test (*p* = 0.939) showed similarity between these results. In CLA, the frequency of alcohol consumption was also high (36.39%), and about 41.31% of the population reported no alcohol consumption. Blood Pb levels between alcohol consumers (33.88 μg·L^−1^) and non-consumers (31.73 μg·L^−1^) were similar according to the Kruskal–Wallis test (*p* = 0.939). Therefore, the results in both districts showed that alcohol consumption was not a major factor for increased blood Pb levels, even though in DMN levels were about nine times higher than in CLA.

People who reported using hair dye in DMN composed 22.22% of the population surveyed (Table A2). In this district, the Kruskal–Wallis test showed no statistically significant difference (*p* = 0.163) between mean Pb levels between users (302.20 μg·L^−1^) and non-users (287.60 μg·L^−1^). In CLA, the number of users of hair dye was higher (38.03%), but there was no statistically significant difference (*p* = 0.737) in blood Pb levels of users (35.75 μg·L^−1^) and non-users (31.31 μg·L^−1^). Therefore, the results in both districts showed that the use of hair dye was not an important factor for increased blood Pb levels, even though levels in DMN users were about nine times higher than in CLA users.

In DMN, the majority of the population (93.93%) used groundwater (wells, Amazon, and tubular type) as a form of caption for human consumption and, in this study, it was not possible to analyze Pb levels in the drinking water. 

### 3.2. Pb Blood Levels and Diet

Table A3 shows the mean blood Pb concentrations associated with food consumption (dietary variables: red meat, fish, chicken, vegetables, fruits, and canned foods) among individuals residing in DMN or CLA districts. The questions required answers on how many times a day the food was consumed.

Blood Pb levels associated with dietary food frequency did not show a decline among the study population in any of the categorical variables for the frequency of food consumed in both DMN and CLA districts.

### 3.3. Frequency of Blood Pb Levels by Age 

**Figure A4:**
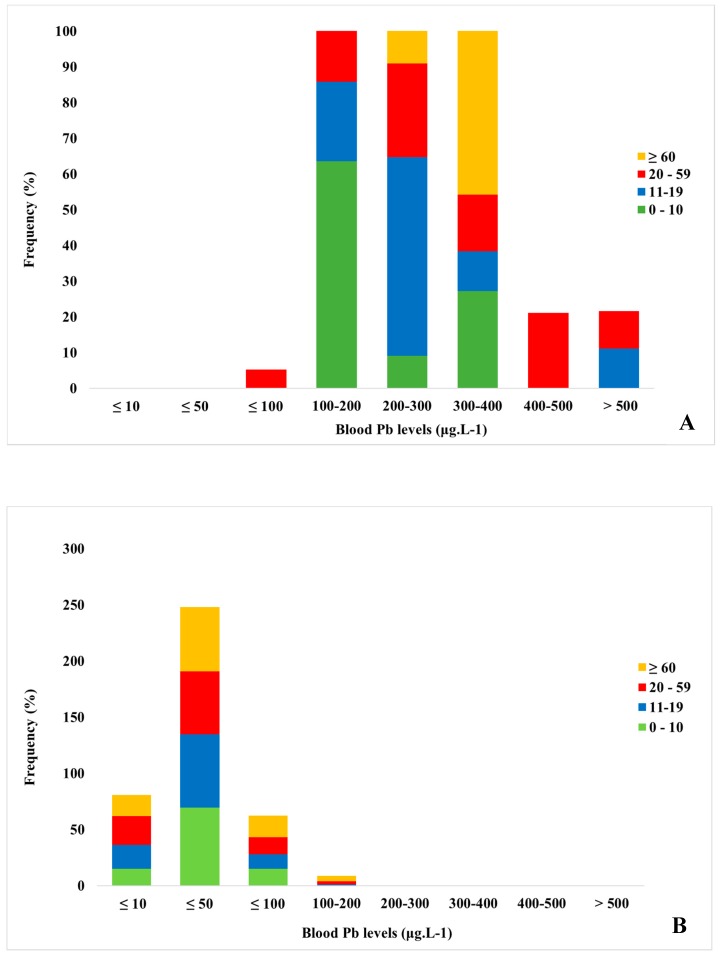
(**A**) Frequency of lead (Pb) blood levels based on the age range in the district of Dom Manuel; (**B**) frequency of blood levels of Pb based on the age range in the district of Laranjal, State of Pará, Brazil.

Figure A4A,B show the frequency of blood Pb levels by age group in both the DMN and the CLA populations. Figure A4A shows that all children evaluated in DMN, aged 0 to 10 years, had blood Pb levels above the tolerable limit recommended by the World Health Organization (WHO; 50 μg·L^−1^) for this age group [9]. Among these children, 27.27% presented with levels in the range 300 to 400 μg·L^−1^, about 6–8 times more than the recommended limit. This pattern was very different from that observed in Figure A4B for the same age group in CLA. In CLA, 72.22% of children had blood Pb levels below 50 μg·L^−1^, and only a small group of 22% had levels ranging between 50 and 100 μg·L^−1^. Therefore, these high frequencies of blood Pb levels among DMN children are of concern because of the high degree of environmental exposure to this contaminant.

This observation was similar for the other age groups including young people, adults, and the elderly. All young and old individuals had blood Pb levels above the WHO tolerable limit for the adult population (100 μg·L^−1^). Among adults, only 5.26% had Pb levels below the tolerable limit. In total, 11.11% of young people, 26.32% of adults, and 50% of the elderly had levels in the range of 300 to 500 μg·L^−1^, i.e., about 3–5 times the limit recommended by WHO. However, it should be noted that 10% of young people and 10.53% of adults had levels above 500 μg·L^−1^, i.e., these individuals had blood Pb levels five times above the tolerable limit [10]. In contrast, in CLA, only 1.25% of young people, 2.82% of adults, and 4.76% of the elderly had levels ranging from 100 to 200 μg·L^−1^. These small variations in CLA may be associated with smoking. In CLA, 18.03% of the population declared being smokers. Therefore, young people, adults, and the elderly of DMN also had a high standard of environmental exposure to Pb. These data confirm that the elderly group is also more exposed to this contaminant.

### 3.4. Mean Concentration of Pb in Individuals from Dom Manuel and Laranjal, and other Countries

**Figure A5:**
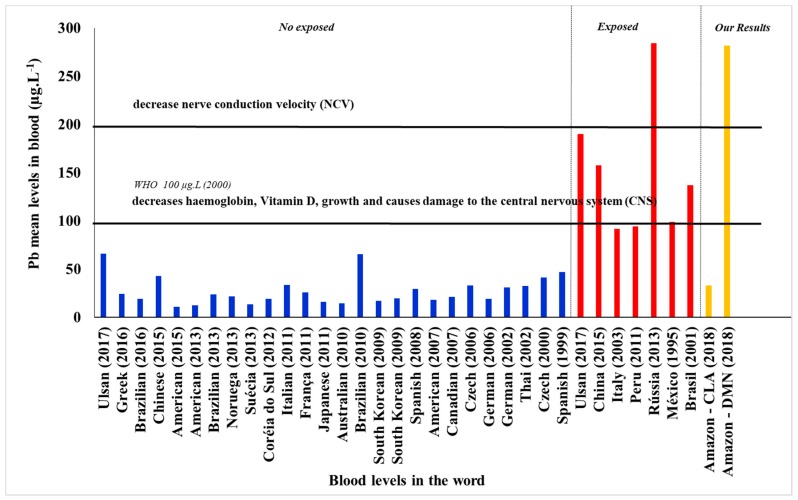
Mean levels of blood Pb in individuals living in the districts of Dom Manuel and Laranjal, and in individuals from other countries.

Figure A5 shows the mean blood Pb concentrations of individuals residing in the DMN and CLA districts in the Pará State, Brazil, compared to levels of Pb found in residents of other countries, based on findings from studies conducted in industrial areas, as well as occupational exposure studies, such as the Russian study. In the non-exposed group, the mean blood Pb levels found in CLA (32.77 μg·L^−1^) were similar to the range found in the literature, globally. In relation to exposed individuals, the mean blood Pb level found in DMN (281.60 μg·L^−1^) was higher than in other countries with the exception of the study conducted in Russia, which showed a geometric mean of 284 μg·L^−1^, slightly higher than that found in DMN. Figure A5 also shows that mean levels of Pb above 100 μg·L^−1^ would be harmful to human health and could possibly cause damage to the central nervous system [11].

## 4. Discussion

To compare the effects of the mean blood Pb levels found in the DMN and CLA (control) districts, a search of the literature for the mean blood Pb levels reported in studies from several countries, including Brazil, was performed. Among individuals exposed to the industrialized environment in a region of China, a study reported that the mean Pb level was 157.47 μg·L^−1^ [12]. 

In the present study, the mean DMN community Pb level (281.60 μg·L^−1^) was five-fold higher than the reference level found in Brazil and other regions of the world. In a study conducted among individuals residing in an area free of industrial activities, in the suburbs of Beijing, various studies and assessments on the exposure of this population showed that the normal value for blood Pb was 42.6 μg·L^−1^ [11]. Therefore, the Pb level found in the CLA district (control area) in the present study was within the reference standards at global level for the control area. The mean Pb level in CLA was 32.77 μg·L^−1^. In the study conducted in Beijing, the authors showed the average blood Pb values for other countries, such as the Americas (12.3 μg·L^−1^), Australia (14.5 μg·L^−1^), Brazil (65.4 μg·L^−1^), Canada (21.3 μg·L^−1^), Italy (33.4 μg·L^−1^), and Korea (19.1 μg·L^−1^) [11].

Blood Pb levels depends on many factors including epidemiological variables such as gender, age, and diet, which may influence blood Pb concentration [13]. In this study, blood Pb levels showed trends in relation to both gender and age group. 

The association with gender (women) may be explained by the presence of bone remodeling in women which is constant during the gestational period, breastfeeding, and up to the postpartum period [14]. However, since the gestational, breastfeeding, and postpartum periods were not variables included in our epidemiological form, it is not possible to know the exact state of these individuals at the time of biological sample collection. The association with gender may also be related to the domestic tasks performed by women in the DMN district, because the district is next to one of the sources of contamination by Pb, whereas men, by going to work, stay in their residences for shorter periods of time than the women. Because this was an area chosen as control (CLA), the presence of Pb can be explained by absorption through different exposure routes such as water, soil, contaminated food, and air [15]. 

Blood Pb levels are higher in adult and elderly individuals because it is an element that bioaccumulates in the human body and is eliminated slowly and gradually. Pb absorption and elimination depends on age, gender, form of absorption of the metal, chemical form of the metal, and form of exposure, among other factors that can contribute to the rise of Pb concentration in human blood [16,17,18]. In this study, Pb levels showed a similar pattern in DMN, with higher Pb levels in elderly adults ≥60 years (317.40 μg·L^−1^), and a constant Pb blood level in the other age groups. This indicates that, in this area, individuals are prone to environmental exposure to Pb through air, soil, and ingestion of contaminated food [11]. The mean Pb level in DMN was 12-fold higher than the Pb levels found in the Metropolitan Region of São Paulo (23.70 μg·L^−1^), one of the most industrialized regions in Brazil that uses public samples in epidemiological studies to monitor the concentration levels of several metallic elements [19].

In CLA, despite this community not being characterized as a control population, the blood Pb level also was higher in the elderly population (≥60 years), confirming previous studies [20]. In DMN, this can be explained by the low educational level of the population, where only 3% of the population had higher or professional schooling [21,22]. It is possible that individuals in this community would have fewer job opportunities and may have spent more time being exposed to Pb in their homes. In the CLA community, higher and professional schooling occurred in more than 50% of the population; however, because it was a control population, blood levels of Pb were considered acceptable [20,21]. 

In CLA, the level of Pb in the blood was higher for illiterate people (48.10 μg·L^−1^); however, this value corresponded solely to one single young individual. Because CLA was a control area in this study, it is worth noting that it was distant from industries and that any Pb found in the blood of these individuals may be associated with occupational factors instead, such as cigarette smoking and alcohol consumption, as well as environmental exposure through water, soil, air, contaminated food, and through embedded exposure status (industrial or non-industrial) [23].

The highest Pb levels were found in the blood of DMN individuals who were not aware of the time they lived in that area. However, in all the categorical variables established to express the period of residence (years), they showed high levels of Pb (307.10 μg·L^−1^), highlighting the relationship between high Pb levels in individuals who lived in the district for ≥10 years. This result suggests that environmental media such as soil, air, dust, and others may have excess Pb, resulting in environmental exposure because of the various industries existing in the community. This finding is similar to a study conducted in Korea where the authors analyzed the blood of individuals in both industrial and control areas. The authors concluded that individuals residing in industrial areas had higher blood Pb levels than those who resided in areas distant to the industries [24].

Blood Pb levels in CLA were higher in individuals who lived in the area for ≥10 years (34.06 μg·L^−1^). It is noteworthy that the CLA district underwent a process of spatial reorganization. At the time of the installation of the industrial pole in Barcarena, CLA was one of the residential areas where some of the companies were located before relocation by the Federal Government to their current location [25]. Because it is a control area, Pb results found may be associated with absorption from regional background levels. Due to the Pb biogeochemical cycle, this element may be present in food, drinking water, and household dust. According to a study carried out in Barcarena, CLA is a neighborhood with a characteristic dwelling, where the majority of the residences have walls painted with colored paint, the streets do not have asphalt, and there is no sanitary sewage, all factors that may contribute to circulating Pb levels in the region [26].

In CLA (control), blood Pb levels were higher, at 39.90 μg·L^−1^, for individuals who smoked [22]. Compared to the control area, the blood Pb level found in this population may be related to the predominance of adult smokers in those aged between 20 and 59 years, where the accumulation of Pb in mineralized tissues causes individuals, even if not directly exposed, to suffer from the mobilization of Pb from the mineralized tissues into the bloodstream.

A study conducted in individuals who consumed alcoholic beverages showed that their blood Pb level was higher regardless of gender. However, excessive alcohol consumption had a significant increase in blood Pb levels predominantly in men [27]. In the DMN district, the Pb blood level was higher for individuals who did not consume alcohol (368.30 μg·L^−1^), indicating that blood Pb levels in the population of DMN were not associated with alcohol consumption. Therefore, these results show that environmental exposure to Pb predominates in this district. Previous studies showed that Pb tends to accumulate in the mineralized tissues re-remobilize to the blood. This high Pb concentration in the blood occurs mainly in adults and the elderly.

The Pb levels found in blood samples in DMN were elevated regardless of whether people dyed their hair. It is known that most capillary products contain significant concentrations of elements that are toxic and harmful to human health. In CLA, Pb levels were higher in individuals using hair dye, reaching 35.75 μg·L^−1^. These results may due to other different routes of exposure to Pb. This occurs through the presence of Pb in the environment, such as water, soil, air, dust, and contaminated food.

The Pb levels in blood in the DMN were higher (281.60 μg·L^−1^) for individuals using water from wells, the only source of water supply for this district. In some studies, the main routes of environmental exposure were through groundwater, food, air pollution, suspended dust, and contaminated soil [28]. Therefore, it cannot be ruled out that groundwater consumption may be one of the routes of Pb exposure in DMN.

In CLA, blood Pb levels were higher (43.42 μg·L^−1^) in individuals who used the distribution network as the source of water supply. The Pb levels in an area with no industries can be related to pipes used in the distribution network, often made of metal that undergoes corrosion over time and releases some constituent materials of its composition (Pb) in places where the water is being collected for distribution to the network. As such, these individuals experience occupational exposure indirectly.

Some studies recommended the treatment of water before human consumption. In DMN, Pb levels were higher when subjects performed the pre-consumption water treatment step, reaching 333.20 μg·L^−1^. This is a form of primary treatment for the removal of the solid fraction (sand and pieces of objects, among others), since the water consumed in the district was from the wells typical of the Amazon. One method to prevent this would be to more thoroughly evaluate ways of treating the water consumed in the community, and to verify the possibility of removing metallic contaminants. A study carried out in the districts of Vila do Conde and Maranhão, located in the municipalities of Barcarena and Abaetetuba, respectively, showed that the water consumed in these places did not receive any type of treatment. The water was dammed, channeled, and distributed to the population for human consumption. It also showed that the aquifers that supplied both districts were contaminated by domestic and industrial effluents [29].

In CLA, the highest blood Pb levels were found in individuals who used boiling as a form of water treatment pre-consumption (84.40 μg·L^−1^). Although the CLA district had two sources of water supply, the most used were the Amazonas and tubular type. The results shown for CLA can be explained by the type of treatment performed before consumption, with possible occupational exposure, as well as smoking and alcohol consumption. Pb contamination of district wells can also occur through the mobilization of soil metal to the underground aquifer that supplies the wells in the region [29,30].

Mean blood Pb levels in DMN subjects, when associated with daily consumption of red meat, fish, chicken, vegetables, fruits, and canned food (Table A3), were always higher than the reference levels for unexposed populations [20]. The Kruskal–Wallis test showed no significant difference (*p* > 0.05) in blood Pb levels in the DMN population when these results were stratified by a higher daily intake of fish (*p* = 0.800), vegetables (*p* = 0.088), and fruits (0.150). The individuals in this population usually consumed fish, vegetables, and fruits that were acquired in the regional rivers (fish) or through family farming (vegetables and fruits). Therefore, these data indicate that ingestion of these foods would not be the main route of Pb exposure in DMN. However, there were significant differences (*p* ≤ 0.005) associated with higher consumption of red meat (*p* = 0.036), chicken (0.003), or canned food (0.038). Data on daily consumption of red meat and chicken showed that blood Pb levels increased (378.00 and 408.00, respectively) for those individuals who ate these products three times per day. In contrast, higher canned food consumption decreased the blood Pb levels (163.00) of these individuals. In this study, Pb levels were not evaluated in the food, but data were indicative of the need to deepen this knowledge in order to understand the routes of Pb exposure in DMN, mainly due to the fact that rearing of animals like chicken is frequent in this district. 

For neurological, metabolic, and behavioral reasons, children are more vulnerable than adults to the effects of the toxic action of lead. Epidemiological studies showed that lead is associated with neurobehavioral deficits in children. The Pb level is a widely used parameter for assessing occupational and environmental levels for exposed population groups. Pb toxicity generates clear clinical effects or biochemical effects. During childhood, Pb exposure can have permanent effects such as a lower intelligence quotient, cognitive deficiency, irritability, and attention deficit. Furthermore, play activities may decrease over several weeks. Throughout a child’s development, the nervous system can be adversely affected if Pb levels are below 10 μg·L^−1^. Above this value, Pb levels of 20 μg·L^−1^ result in changes in the nerve conduction velocity, at 40 μg·L^−1^, it affects hemoglobin synthesis (Hg), and, at 50 μg·L^−1^, there can be symptoms such as abdominal cramps, neuropathy, encephalopathy, anemia, nephropathy, and dizziness [31].

Among adults, nervous system damage is reported in all Pb exposure studies, and symptoms of encephalopathy are reported such as headaches, loss of memory, loss of concentration and attention to everyday tasks, mood swings, irritability, depression, and insomnia (or excessive drowsiness) [9]. However, children would be a vulnerable group especially in areas where the soil is contaminated, as primary contact occurs in various activities such as recreation. In this group, gastrointestinal absorption may occur and may cause damage to organs of the digestive and urinary systems.

Although some age groups in the CLA district had Pb levels higher than 10 μg·L^−1^, where Pb symptoms begin to appear, the Pb levels found in CLA were similar to those found in the literature for non-exposed population groups.

## 5. Conclusions

The results in this study showed that individuals in DMN had elevated blood Pb levels, irrespective of age group and gender, with high concentrations of Pb seen in the most vulnerable groups such as children and the elderly. In relation to diet, there was no evidence of any influence of diet on Pb in the blood or any indication that there was a correlation between the two. The statistical and significant differences found in Pb blood levels in the DMN district were characteristic of environmental exposure to Pb, and individuals that were considered to be smokers were typically more exposed to Pb. However, further studies are needed to establish which exposure to Pb in the environment has high effects. This may occur prior to contact with the human organism, since, in the DMN district, there are currently three industrial activities. These activities range from industries developing petroleum products such as the production and storage of petcoke and mineral coal, the production and marketing of manganese sintering, and storage and export of kaolin ore. In CLA, the results of Pb levels in the blood point to it being a control area due to its distant location from the industrial areas and because of the residential characteristics, a fact that could be proven according to other results obtained from international literature. However, further studies are needed to further understand the route of exposure to Pb in the environment and to identify possible sources of environmental and/or occupational exposure in this area.

## Appendix A

**Table A1:** Mean levels of the total blood lead (Pb) in the districts of Dom Manuel and Laranjal, State of Pará, Brazil, 2012.

Variables	Dom Manuel	Laranjal
*N*	41	294
*N* *	4	11
Average	281.60	32.77
Median	259.60	29.44
Standard deviation	120.90	26.85
Maximum	570.80	171.78
Minimum	98.73	0.07

*N*: number in sample; *N* *: number missing.

**Table A2:** Mean Pb levels in the blood, and the demographics and lifestyle of the individuals of Dom Manuel and Laranjal, State of Pará, Brazil, 2012.

Districts								
Variables	Dom Manuel (*N* = 45)				Laranjal (*N* = 305)			
	*N* (%)	Pb (µg·L^−1^)	*N* *	* *p*	*N* (%)	Pb (µg·L^−1^)	*N* *	* *p*
**Gender**								
Male	23 (51.11)	290.40	3	0.714	129 (41.96)	35.71	6	0.113
Female	19 (42.22)	271.40	1		167 (54.75)	30.51	3	
**Age group (years)**								
0–10	11 (24.44)	221.40	1	0.149	47 (15.40)	34.46	5	0.823
11–19	9 (20.00)	284.10	1		82 (26.88)	33.38	0	
20–59	19 (42.22)	311.40	0		144 (47.21)	31.53	3	
≥60	3 (4.44)	317.40	1		23 (7.54)	34.98	1	
**Education**								
Illiterate	-	-	-	0.266	1 (0.32)	48.10	0	0.008
Fundamental	27 (60.00)	311.00	2		124 (40.65)	32.50	3	
Medium	1 (2.22)	199.72	0		23 (7.54)	23.97	0	
Professional	2 (4.44)	153.50	0		48 (15.73)	36.43	0	
Higher	1 (2.22)	175.81	0		59 (19.34)	29.74	3	
Special	1 (2.22)	271.54	0		4 (1.31)	6.31	0	
NI	10 (20.00)	243.70	1		37 (12.13)	41.68	3	
**Residence time (years)**								
≤5	11 (24.44)	271.30	0	0.174	46 (15.08)	30.93	4	0.130
5–10	11 (24.44)	231.30	1		60 (19.67)	30.12	3	
≥10	17 (35.55)	307.10	1		190 (61.96)	34.06	2	
NI	3 (6.66)	367.10	1		-	-	-	
**Use of cigarettes**								
Yes	6 (11.11)	418.00	1	0.045	55 (18.03)	39.90	2	0.021
No	16 (35.55)	278.90	1		182 (59.67)	30.26	2	
NI	20 (44.44)	249.60	1		59 (19.34)	33.98	5	
**Consumption of alcoholic beverage**								
Yes	16 (35.55)	294.40	3	0.096	111 (36.39)	33.88	4	0.939
No	5 (11.11)	368.30	0		126 (41.31)	31.73	0	
NI	20 (44.44)	249.60	1		59 (19.34)	32.91	5	
**Hair dye**								
Yes	11 (22.22)	302.20	1	0.163	116 (38.03)	35.75	2	0.737
No	27 (60.00)	287.60	2		154 (50.49)	31.31	2	
NI	4 (8.88)	189.10	0		26 (8.52)	28.07	5	
**Water source**								
Well	42 (93.33)	281.60	3	0.125	216 (70.81)	28.81	4	0.001
General network	-	-	-		80 (26.22)	43.42	5	
Water treatment								
Mineral water	4 (8.88)	220.40	0	0.352	64 (20.98)	30.68	0	0.001
Coagulation	8 (17.77)	333.20	1		12 (3.93)	30.65	2	
Boil	6 (13.33)	290.70	0		4 (1.31)	84.40	0	
Hypochlorite	8 (17.77)	259.30	1		165 (54.09)	33.23	2	
Filters	-	-	-		35 (11.47)	29.78	5	
Do not try	16 (35.55)	282.00	1		16 (5.24)	40.92	0	

*N* *: number missing; NI: not informed; *N*: number in sample; (%): frequency; * *p*: statistical difference between blood Pb levels, through the Kruskal–Wallis test.

**Table A3:** Levels of blood Pb associated with the diet of individuals living in the districts of Dom Manuel and Laranjal, State of Pará, Brazil, 2012.

Variables	Districts							
	Dom Manuel (*N* = 45)				Laranjal (*N* = 305)			
	*N* (%)	Pb (µg·L^−1^)	*N* *	* *p*	*N* (%)	Pb (µg·L^−1^)	*N* *	* *p*
**Red meat**								
1× (day)	19 (42.22)	323.10	3	0.036	140 (45.90)	34.13	2	0.916
2× (day)	21 (44.44)	232.50	0		146 (47.86)	32.80	7	
3× (day)	2 (4.44)	378.00	0		-	-	-	
NI	-	-	-		10 (3.27)	28.20	0	
**Fish**								
1× (day)	12 (26.66)	298.60	2	0.800	172 (56.39)	36.47	2	0.367
2× (day)	7 (15.55)	378.00	0		55 (18.03)	35.44	6	
3× (day)	10 (22.22)	281.70	0		1 (0.32)	4.64	0	
NI	13 (28.88)	268.80	1		68 (22.29)	23.86	1	
**Chicken**								
1× (day)	17 (37.77)	337.70	3	0.003	152 (49.83)	34.48	0	0.397
2× (day)	22 (48.88)	253.30	0		138 (45.24)	32.55	9	
3× (day)	3 (6.66)	408.00	0		1 (0.32)	4.64	0	
NI	-	-	-		5 (1.63)	22.06	0	
**Vegetables**								
1× (day)	25 (55.55)	332.60	3	0.088	198 (64.91)	35.48	6	0.010
2× (day)	10 (22.22)	232.40	0		70 (22.95)	25.48	2	
3× (day)	1 (2.22)	199.74	0		3 (0.98)	62.90	0	
NI	6 (13.33)	283.10	0		25 (8.19)	34.07	1	
**Fruits**								
1× (day)	32 (71.11)	307.10	3	0.150	248 (81.31)	31.95	9	0.041
2× (day)	5 (11.11)	249.40	0		34 (11.14)	42.60	0	
3× (day)	5 (11.11)	292.60	0		5 (1.63)	24.20	0	
NI	5 (11.11)	292.60	0		9 (2.95)	39.63	0	
**Canned**								
1× (day)	16 (35.55)	327.10	1	0.038	137 (44.91)	30.42	5	0.874
2× (day)	3 (6.66)	163.00	0		24 (7.86)	27.94	0	
3× (day)	0	-	-		0	-	-	
NI	23 (51.11)	296.30	2		135 (44.26)	37.12	4	

*N* *: number missing; NI: not informed; *N*: number in sample; (%): frequency; * *p*: statistical difference between blood Pb levels, through the Kruskal–Wallis test.
